# Developing drought resilience in irrigated agriculture in the face of increasing water scarcity

**DOI:** 10.1007/s10113-017-1116-6

**Published:** 2017-02-08

**Authors:** Dolores Rey, Ian P. Holman, Jerry W. Knox

**Affiliations:** Cranfield Water Science Institute, Cranfield University, Cranfield, Bedford MK43 0AL UK

**Keywords:** Adaptation, Drought management, Farmer, Water resources, UK

## Abstract

In many countries, drought is the natural hazard that causes the greatest agronomic impacts. After recurrent droughts, farmers typically learn from experience and implement changes in management to reduce their future drought risks and impacts. This paper aims to understand how irrigated agriculture in a humid climate has been affected by past droughts and how different actors have adapted their activities and strategies over time to increase their resilience. After examining recent drought episodes from an agroclimatic perspective, information from an online survey was combined with evidence from semi-structured interviews with farmers to assess: drought risk perceptions, impacts of past drought events, management strategies at different scales (regional to farm level) and responses to future risks. Interviews with the water regulatory agency were also conducted to explore their attitudes and decision-making processes during drought events. The results highlight how agricultural drought management strategies evolve over time, including how specific aspects have helped to reduce future drought risks. The importance of adopting a vertically integrated drought management approach in the farming sector coupled with a better understanding of past drought impacts and management options is shown to be crucial for improving decision-making during future drought events.

**Electronic supplementary material:**

The online version of this article (doi:10.1007/s10113-017-1116-6) contains supplementary material, which is available to authorized users.

## Introduction

Climate change combined with population growth, increasing pressure on freshwater resources and greater regulatory demands for environmental protection will all impact on agricultural productivity (Knox et al. 2016); an increase in the magnitude and frequency of extreme events, such as droughts, will exacerbate the problem (Fedoroff et al. 2010; OECD 2010; Jiménez Cisneros et al. 2014; Iglesias and Garrote 2015). Agriculture is one of the sectors that suffers most from the consequences of droughts, which are responsible for the greatest loss of agricultural production in many countries (Wilhite 2007). The impacts of drought on agriculture are becoming an important abiotic stress in temperate and humid regions (Knox et al. 2010a).

A drought is normally defined as a natural hazard caused by a period of abnormally low precipitation. Drought impacts on crop yield and quality depend on numerous factors, including the onset of drought relative to the stage of crop development, the water source reliability, the vulnerability of each crop type to water stress and socio-economic factors. The impact of droughts on food supply is thus a combination of the weather itself and the resilience of the different parts of the food supply chain to those impacts (Benton et al. 2012). There are several definitions of resilience; for the purposes of this study, we have adopted the United Nations definition that refers to “the capacity of systems (ranging from national, local or household economies to businesses and their supply chains) to anticipate, absorb or buffer losses, and to recover” (UN 2015).

As reported by Wreford and Adger (2010), farmers typically learn from previous drought events and adapt their businesses based on their experiences. The actions aimed at reducing drought risks and impacts on farms can be categorised according to their timescale, whether they are aimed at increasing water supply or reducing demand, and their spatial scale of intervention (Iglesias et al. 2009, 2012). However, most recent research on adaptation processes has mainly focussed on agricultural systems in arid and semi-arid regions (e.g. Santos Pereira et al. 2002; Habiba et al. 2012; Wheeler et al. 2013; Kirby et al. 2014). Despite the apparent lower reliance on irrigation in temperate or humid regions, it can be a highly productive use of water. For example, although irrigation in England and Wales typically represents only 1% of water use nationally and is supplemental to rainfall (Knox et al. 2010b; 2013), the financial benefits of irrigation in a dry year at the farm level are substantial (Rey et al. 2016).

Despite being a humid region, drought is an inherent feature of the UK climate. The 1975–1976 drought is widely regarded as being the most severe (Royal Society 1978; Burke et al. 2010). However, recent drought events have also caused severe regional impacts on agricultural production. For example, the 2010–2012 drought caused an estimated £400 million in farming losses (Anglian Water, University of Cambridge 2013). A changing climate is projected to lead to an increase in the frequency of hotter and drier summers, and short-duration droughts with major consequences on crop production (Hulme et al. 2002; EA 2013).

In the UK, irrigated agriculture is predominantly concentrated in eastern England. It accounts for over half (60%) the total irrigated area and volume of water used for irrigation (57%) nationally, with the majority of production located in catchments classified as being either over-abstracted or over-licenced (Hess et al. 2010). To secure sufficient environmental river flows and meet rising water demands (Weatherhead et al. 2015), increasing water scarcity is likely to compound the drought challenges faced by irrigated agriculture in this region. Whilst much attention has been paid to arid regions, this paper aims to understand how agricultural drought management in a humid climate, ranging from farm to catchment scales, has adapted in response to past droughts and increasing water scarcity and the extent to which this might have influenced drought resilience. Through an online survey and interviews with farmers and regulators in eastern England, this research assesses (1) how drought management has evolved over recent decades, (2) how farmers perceive drought risks, (3) their likelihood of being affected in future and (4) what improvements have been implemented in drought management in UK agriculture. A better understanding of these issues will inform future decision-making and thus increase drought resilience. There are also some fundamentally important lessons for other humid or temperate regions internationally.

## Materials and methods

### Case study

**Fig. 1:**
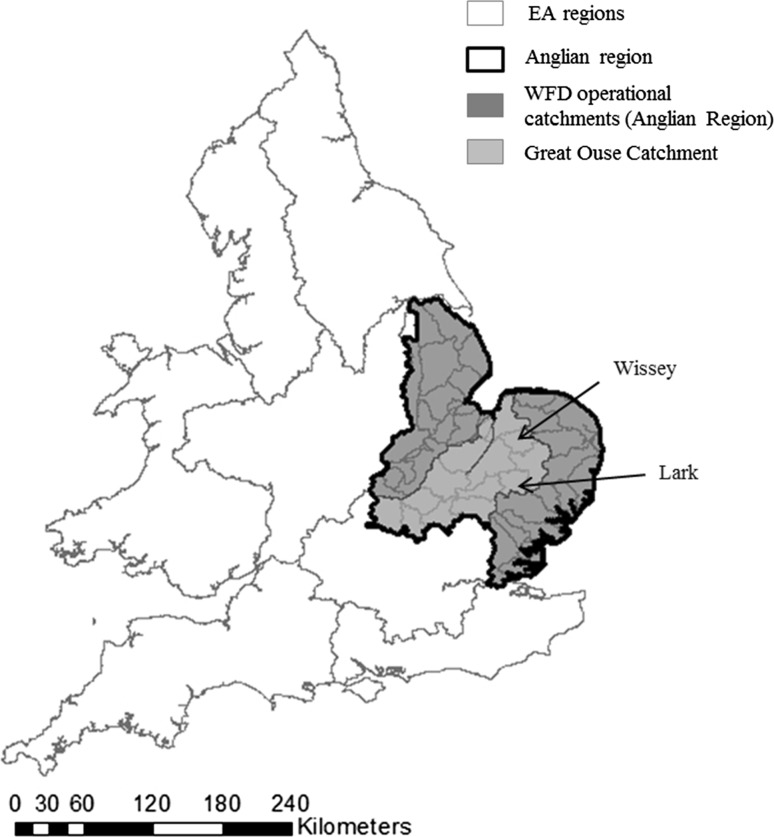
Anglian region of the environment agency and catchments studied

The Anglian region of the Environment Agency covers an area of 27,890 km^2^ (Fig. 1). Due to favourable soils, topography and agroclimate more than half the area is dedicated to agricultural and horticultural production, with high-value irrigated vegetable cropping using 160 Mm^3^ water in a dry year (Weatherhead et al. 2015). Average annual rainfall is 600 mm (less than 70% of the national average) and annual reference evapotranspiration (ETo) averages 530 mm. The Environment Agency (EA) is the water regulatory agency for England and Wales, responsible for environmental protection and water resource allocation. According to climate change projections, the frequency and severity of extreme events will increase in the region, and summers will be drier, affecting water availability when crops need it most and increasing the likelihood of abstraction restrictions (Fowler and Kilsby 2004; Murphy et al. 2009; CCC 2013; EA 2013a). The EA manages water abstraction through a statutory licensing system (EA 2008a). All irrigators using more than 20 m^3^ per day must have an abstraction licence issued for a time-limited period, normally 12 years (EA 2008a). The licence has conditions to protect other water users and the environment. Section 57 of the Water Resources Act 1991^1^ gives the EA powers for emergency variation of licences for irrigation when there has been an exceptional shortage of rainfall or a water scarcity situation, to protect public water supply and secure minimum environmental flows. Abstraction from groundwater would only be restricted if it is likely to affect the flow of an inland water such as a river or stream. Many irrigators were restricted during previous drought episodes with significant associated economic impacts (EA 2011, 2012; Vivid Economics 2013).

### Data collection and analysis

This research combined quantitative agrometeorological data with qualitative evidence gathered from an online survey and semi-structured interviews to analyse how the drought resilience of irrigated agriculture in the Anglian region has evolved over time.

#### Drought severity assessment

The climatic severity of all recent drought events (1975–1976, 1988–1992, 1995–1997, 2003, 2004–2006, 2010–2012) (EA 2006) was assessed using the Standardised Precipitation Index (SPI) drawing on data from the CEH Drought Portal.^2^ The SPI represents the variation in rainfall deficit from the mean in a standardised way. Over short timescales the SPI is closely related to soil moisture conditions. Thus, we used a moving window 3-month SPI. This provided a comparison of precipitation over a specific 3-month period with the precipitation totals from the same 3-month period for all the years included in the historical record (WMO 2012). As important as the severity of the drought, is its timing and onset. We therefore analysed the drought severity for each month for a representative catchment in the region, focusing on those months coinciding with the crop development cycle for the most important irrigated crops. In addition, the maximum potential soil moisture deficit (PSMDmax) was used as an agroclimatic indicator to reflect the relationship between aridity and irrigation need (Knox et al. 1997). PSMDmax values were calculated using a 5 km × 5 km gridded monthly climatic data set from the UK Meteorological Office derived from observed historical weather data (Perry and Hollis 2004). ETo was calculated applying the FAO Penman–Monteith combination equation (Allen et al. 1998). Using monthly rainfall (*P*_*t*_) and reference evapotranspiration (ETo_*t*_) data, annual PSMDmax is identified from the PSMD (mm) for each month (*t*), calculated as the following:

$${\text{PSMD}}_{t} = {\text{PSMD}}_{t - 1} + {\text{ETo}}_{t} - P_{t}$$

In months where *P*_*t*_ > (PSMD_*t*-1_ + ETo_*t*_), any initial soil moisture deficit is filled and hence PSMD_*t*_ = 0.

#### Online survey

An online survey was sent to all members of the UK Irrigation Association (UKIA) in December 2014, which consisted of 20 closed-ended questions, categorised into four sections: (1) baseline farm information, (2) drought impacts, (3) drought management and responses and (4) drought risk perception. Although data were collected nationally, this paper focuses on the Anglian region, as it is the most important irrigated area in the UK. The farms (*n* = 26) were heterogeneous in terms of their farm size (50–4400 ha), the proportion of the farm area that could be irrigated (40–100%), the water resources available for irrigation, the types of abstraction licence held and the irrigation methods (Table 1). Although the overall sample was relatively small, the farms represented a significant proportion (62%) of the total irrigated area in the region, making it a representative sample of irrigated agriculture in the area.

**Table 1:** Summary statistics for growers involved in the survey

Descriptor	Categories	Farmers (*n*)	%
Farm size (ha)	0–200	5	19.2
	200–500	5	19.2
	500–1000	5	19.2
	1000–2000	5	19.2
	>2000	6	23.1
Irrigated crops	Maincrop potatoes (irrigated)	21	80.8
	Early potatoes (irrigated)	17	65.4
	Vegetables	20	76.9
	Sugar beet	11	42.3
	Cereals	14	53.8
	Grass	2	7.7
	Small fruit	1	3.8
	Orchard fruit	2	7.7
Water source	Surface water	24	92.3
	Groundwater	26	100.0
	Public mains supply	1	3.8
	Rainwater harvesting	2	7.7
	Water reuse	2	7.7
Type of licence	All year abstraction	10	38.5
	Summer-only abstraction	21	80.8
	Winter-only abstraction	11	42.3
Irrigation method	Static or hand-moved sprinklers, spray lines	2	7.7
	Hose reels with rain gun	24	92.3
	Hose reels with boom	15	57.7
	Centre pivot or linear move	4	15.4
	Trickle or drip	2	7.7
Final destination of production	Local farmers’ market	3	11.5
	Processing	24	92.3
	Supermarket	22	84.6
	Export	13	50.0
	Other	10	38.5

#### Semi-structured interviews

The participants from the survey were then invited for an interview. Fifteen farmers were interviewed (nine face to face and six by phone) between February 2015 and March 2016. Questions were open-ended to derive an in-depth understanding of decision processes at the farm level to cope with droughts, and to elicit information on farmer memories from past drought events. The interviews were recorded, transcribed and coded using a template analysis approach (King 1998). This involved the development of a coding template to summarise important themes in the data and organising them in a meaningful way. The analysis started with a set of a priori codes to identify relevant themes. During the coding phase, one or more codes were assigned to each relevant piece of text. During reading of the transcripts, new codes arose and some a priori codes were removed or merged with others, as needed. This method facilitated the interpretation of the qualitative data contained in the interviews (the final version of the thematic coding template is available as supplementary material).

#### Interviews with water regulatory staff

We also interviewed two EA drought coordinators by phone (representing two of the three administrative regions within the Anglian region). The questions related to their specific roles within the agency during recent drought events, their memories from previous drought episodes and the regulatory actions and management responses that have been established, including implementation of abstraction restrictions.

To demonstrate through our empirical analysis that drought resilience has increased in irrigated agriculture in the Anglian region, we focus our attention on the following issues and how they have evolved over the period under study: (1) droughts impacts on crop yield; (2) range of drought management strategies applied at the farm level (both coping strategies and long-term planning); (3) collaboration amongst farmers and between farmers and the regulator.

## Results

### Agrometeorological perspective

Table 2 shows the drought severity based on the 3-month SPI value for each month and the PSMD_max_, for recent historical drought events for the Great Ouse catchment (Fig. 1), a large catchment where irrigation is concentrated, and where there is a high degree of water resource stress (EA 2008b). The period 1975–1976 is often remembered as being the most severe drought event in the UK. However, the period 1995–1997 was the driest on record in the south and east of England (EA 2012) when most months recorded an SPI value < 2 (extreme drought) and had the highest proportion of dry months over the period studied; whilst the highest PSMD_max_ value was attained during the 1988–1992 drought. As shown in Table 2, the Anglian region was also affected by several multi-year droughts which, although having a lower 3-month SPI value that the other drought events mentioned above, still had the potential for more severe impacts due to difficulties in winter reservoir filling or for the recharge of groundwater and/or river flow levels.

**Table 2:**
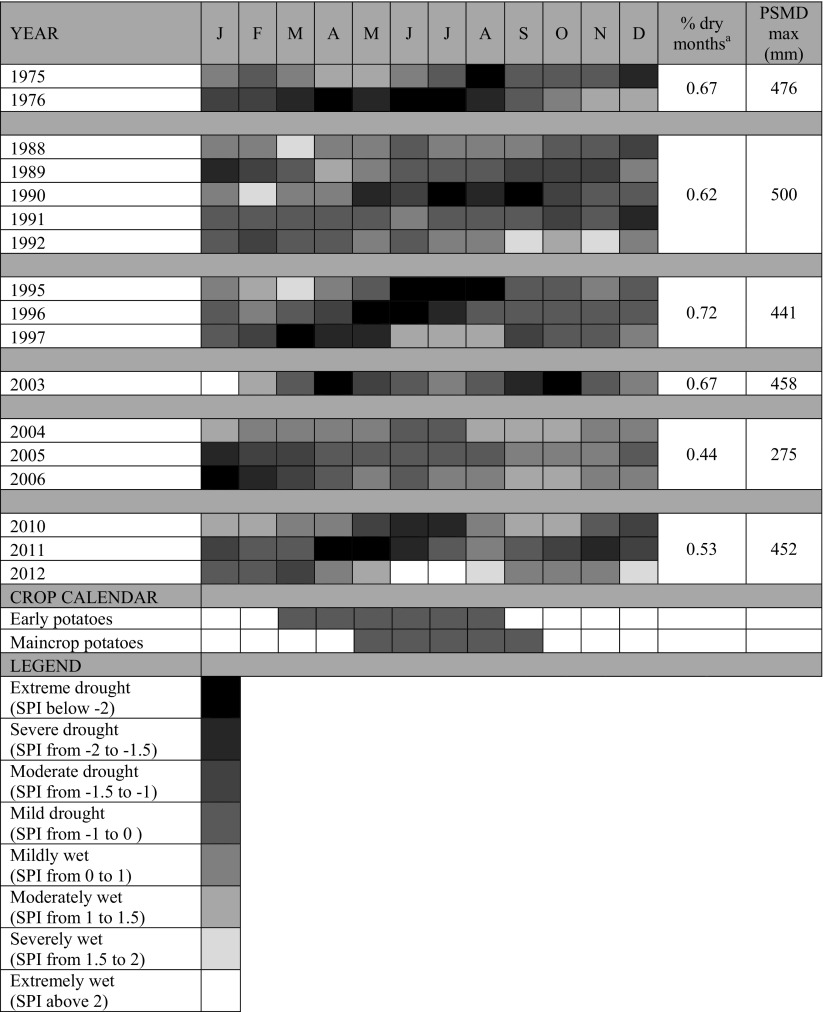
Drought severity based on 3-month standardised precipitation index (SPI) and annual maximum potential soil moisture deficit (PSMD_max_) for recent historical drought events for the Great Ouse catchment, and the cropping calendar for the most important irrigated crops in the area. *Source*: Adapted from CEH drought portal

^a^Calculated as the proportion of months in the drought period with a negative SPI value

### Farmers’ perception of drought and drought risk

Depending on the specific circumstances of each farm, the same drought could have very different types and level of impact between individual farmers. During the interviews, some respondents discussed what drought meant for their business, and defined it in differing ways:

“So, this whole thing is about distribution of rainfall patterns, isn’t it?”

“It appears that 1 year in 5 rainfall drops to 175 mm or below.”

“Water scarcity is wherever there is drought, and the other way around. One becomes the other. It is a risk to your business.”

“On this sort of [sandy] soil, the word drought is not always used that much because we have to manage water so actively anyway.”

Other farmers referred to drought as the situation when they face problems, mainly relating to the duration and timing of the drought event. For example, a period of three weeks without rain was considered by one farmer as a drought, whilst another farmer reported having water availability problems after 6 weeks with very low rainfall in May and June. Some farmers stated that the worst scenario was actually a dry summer following a dry winter, as water reserves would not be replenished and the risks of abstraction restriction were therefore much higher. These differences amongst farmers highlight the complexity of this natural hazard and how dependant their definition is on each farm’s specific circumstances.

Farmers were also asked to rate drought risk for their business on a scale from 0 (not important) to 10 (extremely important). For 18 survey respondents, drought risk was considered a very important business risk (8–10). Only two farmers did not consider drought to be an important risk (2) but, in both cases, they had sufficient licenced volume (and therefore sufficient headroom^3^) to meet crop needs and had never suffered mandatory abstraction restrictions. Interestingly, they view drought as an opportunity rather than a risk with scope to benefit from their competitive advantage over rain-fed production systems and/or other irrigators.

Nearly half (46%) of the farmers surveyed believed that it was “highly likely” and a third (31%) “likely” that droughts would become more frequent in future. During the interviews, some farmers highlighted that any future increase in the frequency and severity of water availability problems not only would be related to weather patterns, but would also be due to an increase in water demand and/or from new water regulation that could reduce their licenced headroom.^4^ Three interviewees believed that droughts were not likely to become more frequent in the future. They reported low–medium impacts during previous drought episodes and had not suffered mandatory abstraction restrictions.

### Impacts of past drought events

#### Irrigation abstraction restrictions

**Fig. 2:**
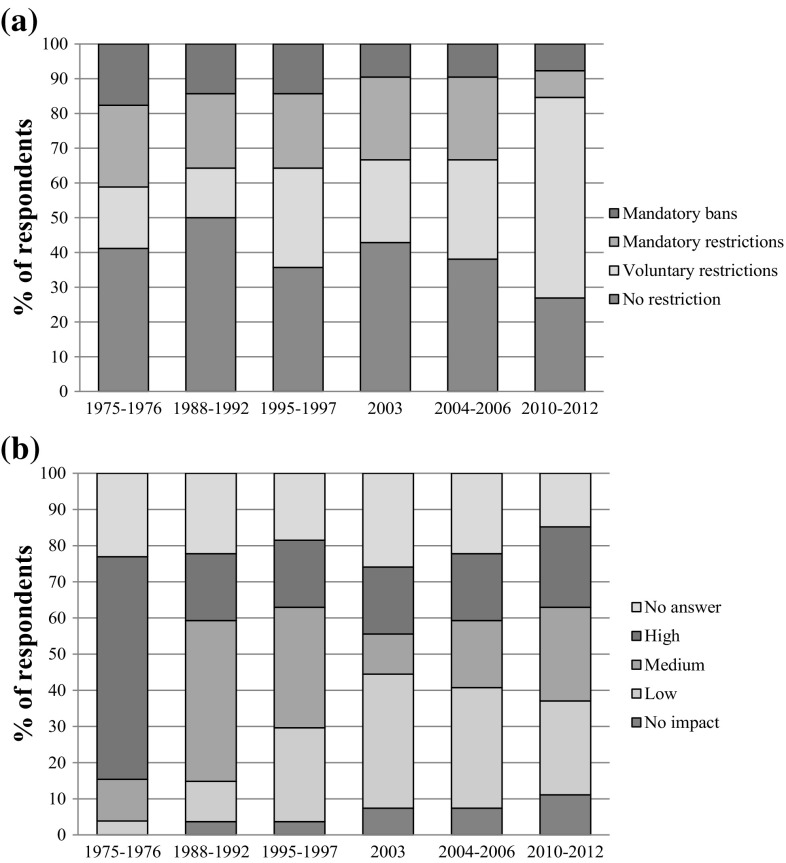
Summary of **a** abstraction restrictions imposed by the water regulatory agency and **b** reported impacts of past drought events on crop production (yield and/or quality) during past drought events derived from farmer survey (*n* = 26)

We asked farmers whether they had been affected by abstraction restrictions imposed by the water regulatory agency (EA) during past drought events, and to indicate whether they were voluntary or mandatory partial restrictions, or total bans (Fig. 2a). Three quarters of survey respondents had been subject to some form of abstraction constraint during all previous drought events, but the analysis revealed a decreasing trend in the proportion of farmers being affected by mandatory bans and mandatory restrictions. Nine participants relied mostly on groundwater, which represented 75–100% of their total irrigation water availability; groundwater abstraction was reported to be seldom restricted by Section 57 regulations.

#### Farmer perceptions of past drought impacts

This research focused on the period from 1975–1976 onwards, as our main source of information was from farmer memories of past drought events. Almost a quarter (23%) of farmers surveyed did not answer the question regarding the impact of the 1976 drought on their production (Fig. 2b), although this proportion was similar to subsequent droughts. The remaining sample of farmers remembered the 1975–1976 droughts as having a medium or high impact on their crops. Since then, there was a generally positive trend in the proportion of farmers that categorised subsequent droughts as having either a low or no impact. This is despite there being little change in the frequency of abstraction restrictions (Fig. 2a) and in the severity of past drought events (Table 2), which could be a sign of the increasing resilience to droughts.

When reflecting on past experiences, it is also important to capture farmer sentiment and opinion on drought. Table 3 summarises the representative comments made by the interviewees about previous drought episodes; it should be recognised that these comments relate to a farm’s situation at that time, which may be very different now due to changes in water management. Nevertheless, based on these data, the irrigated sector and fresh produce supply chain were not well prepared for dealing with water shortages in 1976, leading to severe impacts in Anglian region. The droughts in 2003 and 2004–2006 were not remembered as being “high impact” events. In 2010–2012, although the severity of the drought was reported to be high, some farmers stated that they managed the situation effectively with limited impact. This narrative summarises the evolution of the impact and management of droughts in the region from a farmer perspective and is also useful to compare the comments made by farmers in relation to the 1976 and 2010–2012 droughts. Based on this evidence, farmers felt better prepared and organised in most recent drought episodes, highlighting the increase in resilience within the irrigated agriculture sector.

**Table 3:** Summary of selected farmer comments regarding recent drought events in Anglian region

Drought	Farmer comments
1975–1976	I was farming with my father in Lincolnshire on strong land with good water holding capacity and it ruined most of our crops. Here on this farm with the light land some cereals crops weren’t even hardly worth harvesting
	It was not just the drought; it was the effect on the market. I guess irrigation was not such a big thing, and regulation was not a big thing on water abstraction so the effects of that were more different. And the market has changed a lot since then
	The thing is, at that time, we only had one reservoir of 1 million gallons and we ran out of water in weeks
	We couldn’t irrigate in 1976 here, we were not organized
	I can remember 1976 had a big impact, but growers were still better off because prices compensated for the lack of yield. Of course in 1976 there wasn’t so much product going to the supermarket
	In 1976…yes, fortunately there were no restrictions on the water we could take at that time, S57 did not apply. None of our water resources actually ran out of water physically. So the limiting thing was the machines we had to apply water really at that time
1988–1992	That was high impact of course because that runs up to the formation of Lark Abstractors Group so that was pretty high
	I would suggest those figures [yield] probably fell to 50%
	…We got to the point we couldn’t irrigate some of our crops because the river run dry. So subsequently we invested money in a winter filled reservoir and since then we haven’t really been short of water
	It was a lot of extremely hard work, because those were the days we didn’t have rain guns, all was sprinklers and hand-move sprinklers. It was a long hot summer, we didn’t get 2 inches of rain…
	The boreholes that were closest to the meadows (3 or 4 of them) were effectively shut down
1995–1997	I cannot remember whether we had any restrictions…it certainly wouldn’t have been voluntary, that is for sure. If any, it would have been mandatory
	That was a 2 years drought, with a dry winter in between, so the reservoirs and rivers etc. did not recharge over the winter
	The 1990s generally was a dry decade, drier than average generally I believe, and we wanted to secure our water supply a little bit more because it was coming under pressure, it was being restricted, a critical time…and we needed the reliability of this supply
	…We had severely low flows in the river, low rainfall. It was affecting the biodiversity in the river, so we have lack of oxygen […] fish were dying…There were some fairly drastic measures that were taken to stop that, so there was no abstraction out of the river
2003	2003, I don’t think it was that bad
	If it would be terrible I would have remember, so I don’t think it was…
	About 2003 we changed the way we irrigate, from just irrigating potatoes, we cut the area of potatoes in half and start growing salads and organic salads. So it has been a change in the cropping since then
2004–2006	I don’t remember we have anything in 2003 or 2004–2006
	…We had to alternate the irrigation on surface water. […]. Alternate days were not very useful. Did it affect us? Because we are a mix of surface and groundwater we managed to irrigate every day. And reservoirs…
2010–2012	The yield reduction was marginal because we were able to manage the situation
	The number of conversations that were going on between packers and potato growers around the world to make sure that they do not run out of potatoes… That was happening
	I think the 2011 drought was localized to the East […] I cannot remember how the national yield data (potatoes) looked like but I am fairly confident that it was no decreasing yield across the country…
	We had sufficient warning during the 2012 season not to get in contract situation with any of the irrigated crops
	We were part of the offer of voluntary restriction in 2012–2013 with the EA locally as part of the Lark abstractors group. So there was a voluntary offer to restrict our abstracted volume to 85% of licence. So we were part of that but in the end the weather broke at it rained for nearly all year… so it wasn’t actually restricted
	The only reason why 2012 will not be remembered as the 1976 is because in 1976 it didn’t start raining until the end of August whereas in 2012 it started in June

### Drought management strategies

We can distinguish different types of drought management action based on the spatial scale and time frame. Spatially, the array of actions ranged from farm-scale responses to catchment-scale actions. In relation to timescale, we can differentiate between short-term coping strategies that adapt farm activities to water availability at a point in time within the drought and longer-term strategic business developments designed to manage future drought risks and increase resilience.

#### Short-term coping strategies (farm level)

**Fig. 3:**
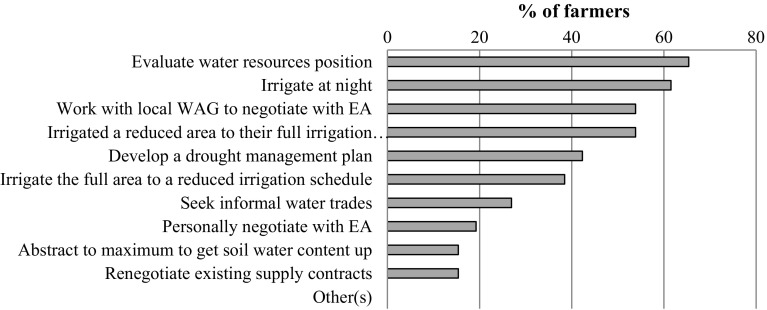
Summary of main strategies implemented by farmers when a drought has been declared and irrigation abstraction restrictions are “likely” (*n* = 26). *EA* environment agency, *WAG* water abstractor group

During a drought, there are various on-farm strategies that could be applied in order to reduce the economic impact and help farm business to meet their contractual obligations (if any). Figure 3 shows the proportion of surveyed farmers using different strategies and Table 4 describes them in more detail, based on the comments during the interviews. These can be broadly classified into three groups: (1) strategies aimed at making best use of available water relative to their own water resource position and infrastructure constraints; (2) liaising with the water regulator (directly or indirectly) to either reduce the likelihood of abstraction restrictions and/or to obtain maximum warning and support from them; and (3) implementing additional coping strategies such as water trades or renegotiating existing contracts.

**Table 4:** Characteristics of the main short-term coping strategies applied by farmers in the study area in response to drought and abstraction restrictions

Coping strategy	Description	Limitations
Evaluate water resource position	To assess how much water is available for the crops and then make a decision about how best to proceed	
Crop prioritization	To prioritise certain crops or varieties based on their drought tolerance and/or economic value	Not suitable for farmers that focus their irrigated production on one main crop
Irrigate reduced area to the full schedule	If there is not enough water to irrigate all the crops, the farmer will only irrigate a certain area/crop based on priorities	This can lead to substantial yield and quality impacts on the remaining crop area
Irrigate full area to a reduced schedule	If there is not enough water to irrigate all the crops, the farmer will irrigate all the crops although the water requirements would be not fully met	Could affect quality, so less suitable for high-value crops (potatoes, vegetables) subject to forward contract commitments
Irrigate at night	Only irrigate at night to reduce ET losses	Irrigation infrastructure could be insufficient to irrigate the full crop area during night hours
Water trading	To trade water with other water abstractors, to obtain extra water during water shortage periods	Administrative licensing process is not straightforward or quick. Several barriers to trade. It needs the approval of the EA

Growers normally applied a combination of strategies (Fig. 3) rather than relying on only one option. For instance, 17 of the 26 survey respondents used four or more strategies during a drought event. Farmers were also asked to identify their two most favoured strategies. They choose (1) working collectively through a local water abstractors group (WAG) to negotiate with the water regulator (EA) (*n* = 7) and (2) developing a drought management plan (*n* = 6) as being most relevant.

Evidence from the interviews suggests that the impact of drought on UK crop prices during and after a drought is not as high as it was a few decades ago due to the increased importance of international markets and a more vertically integrated and developed fresh produce supply chain. Consequently some irrigators in Anglian region enter into fixed-price forward contracts with supermarkets or processors at the beginning of the season to reduce their exposure to price volatility. In these cases, they stated that their decisions during a drought will be driven by prioritising contract commitments when deciding how to share a limited water resource amongst their crops. There may be significant financial penalties if they are unable meet their contractual obligations, and they could risk the renewal of the contract for the following season. For instance, a grower on very sandy soils who grew rain-fed cereals as part of their crop rotation in 2012 stated:

“we had to default on our forward contracts for cereals and it was very costly to buy ourselves out because the market went against us.”

#### Longer-term strategic planning

After being affected by past drought events, most participants made changes in their businesses to increase their resilience to future droughts. The main options undertaken were:

- Development of a drought management plan to establish a protocol for the business in the event of drought (7%);
- Investment in alternative water resources and more efficient irrigation infrastructure (43%). This includes long-term investments to secure water supply (e.g. reservoir construction, multiple abstraction sources, rainwater harvesting), on-farm distribution networks and switching to more efficient irrigation application technologies;
- Modifying crop selection and planting programmes to grow more drought-tolerant or less water-intensive varieties (18%);
- Other strategies (11%), like improving soil management to increase water retention, and adopting collective action through farmer associations such as abstractor groups or producer organisations.

A fifth (20%) of respondents reported they did not change their business management practices to cope with future droughts after the 2010–2012 events. This could be due to the fact that they were not adversely impacted by drought in the past. On the other hand, two-thirds of the farmers interviewed had subsequently invested in winter storage reservoirs to increase the reliability of their summer irrigation water supply (for one farmer, it was their main water source representing 80% of irrigation water on-farm); 20% had considered building a reservoir but had not yet made the investment decision. Another 20% were not considering water storage for different reasons (for example, one farmer believed reservoirs were not the preferred solution because if there was a drought and Section 57 restrictions were enforced there could be additional problems for reservoir filling). Although grants are available to support farm reservoir construction, the main barriers included the high investment cost and uncertainty in how often the reservoir will be used, thus impacting on the investment return.

### Water regulatory agency drought management

The reduction in mandatory abstraction bans (Fig. 2a) is consistent with comments made by a number of farmers about how the water regulator has significantly changed its relationship with the agricultural community from being a “draconian” regulator to having a much more open, transparent and engaged attitude in recent years, with a stronger intent to avoid mandatory abstraction restrictions. Whilst some farmers still view the water regulator as “the police”, the EA has developed a much more proactive approach to communicating with farmers during a drought, through regular meetings, providing information on changing river and aquifer levels, and on prospects for irrigation for the forthcoming growing season. Collectively, these actions have allowed farmers to respond and adapt their management activities to changing water resource conditions with much greater confidence and enabled local water resource planners to work with farmers to provide them with greater flexibility to manage and minimise the drought impacts. The following quotations from farmers highlight these issues:

“Relations with the Agency have improved immeasurably over the last 15–20 years. They are much more ready to talk to abstractors, to discuss the problems, to try to reach solutions that enable them to fulfil the regulatory rules plus give as much flexibility to the abstractors as possible”.

“The EA … they gave us a lot of forward notification. They were forecasting about if we have average rainfall we will need to have this level of restriction…And that was extremely helpful. It gave us the ability to plan our risk…”

The water regulator has also acknowledged during the interviews the critical importance of changing its approach, focusing more on the dialogue with the farming community and the establishment of early lines of communication when a drought appears likely. For example, they now involve farmer representatives in discussions in order to facilitate agreement on voluntary reductions rather than impose mandatory ones later in the season. This approach was successful during the 2010–2012 drought (Fig. 2a) despite its severity (Table 2). Nevertheless, some farmers believe the water regulator should provide better information (e.g. abstraction restriction triggers), could engage more proactively with abstractors in identifying drought responses to balance the needs of agriculture with other users and the environment and should provide more robust evidence of the environmental impact of droughts and the resilience of aquatic ecosystems to justify their decision-making processes regarding implementation of abstraction restriction.

## Discussion

This research aimed to increase our understanding of past drought impacts on irrigated agriculture in eastern England and whether short- and longer-term management strategies were enabling irrigators to become more resilient to droughts in the face of increasing water scarcity. Our findings are based on the combined analysis of agroclimatic data for recent drought events in the Anglian region with an online survey and semi-structured interviews with irrigators. As with any qualitative analysis, this approach has some methodological limitations that need to be recognised. First, our main source of information was from individual memories regarding drought events that happened some decades ago, so inevitably there could be key details that have been forgotten by participants. Secondly, although the thematic coding template allows for a consistent process of retrieving information from the interviews, there is a level of subjectivity in qualitative research that needs to be taken into account. Finally, although the survey participants represent a significant proportion of irrigated agriculture in the region, this sample would not necessarily capture the contrasting range of other views and sentiment expressed by a wider sample from the irrigation community in the Anglian region. Notwithstanding these limitations, the findings do provide highly valuable insights.

According to our analyses, farmers perceive that level of impact of past droughts has decreased over time despite little change in drought severity, as our agroclimatic data analysis clearly showed (Table 2), with the 1975–1976, 1995–1997 and 2010–2012 droughts being the more severe ones in recent history, with some months in the “extreme drought” category, and all of them were multi-annual droughts. This is consistent with farmers’ memories about these drought episodes (Table 3). Farmers also described an increase in the drought management strategies implemented at the farm, increasing the resilience of their businesses to this natural hazard. Wreford and Adger (2010) identified that the deviation from the mean of crop production during past drought events in the UK since the 1970s has decreased over time for potatoes, oilseed rape and wheat. They asserted that the main explanation for this is irrigation. Our study has shown that having supplementary irrigation *per se* does not provide complete resilience to drought, as shown by the proportion of respondents experiencing abstraction restrictions or bans (Fig. 2a) and their significant drought impacts on crop production (Fig. 2b).

However, our results concur with Orson (1999) regarding the importance of both irrigation and water storage. Nearly half (42%) of the farmers surveyed in our study had invested in alternative water sources, like on-farm reservoirs to synchronise abstraction timing with water availability. This finding is similar to that of the NFU (2015) who reported that 50% of respondents in the Lark and Wissey catchments (Fig. 1) have one or more reservoirs. Farms with a larger irrigated area are more likely to have on-farm reservoirs (NFU 2015), which is consistent with the increased specialisation of supplementary irrigation in farm businesses in the last decade, concentrating on larger areas of fewer high-value crops (Morris et al. 2014), a situation that is similar to more drought-prone countries such as USA or Australia (Zilberman et al. 2002; Kirby et al. 2014).

**Fig. 4:**
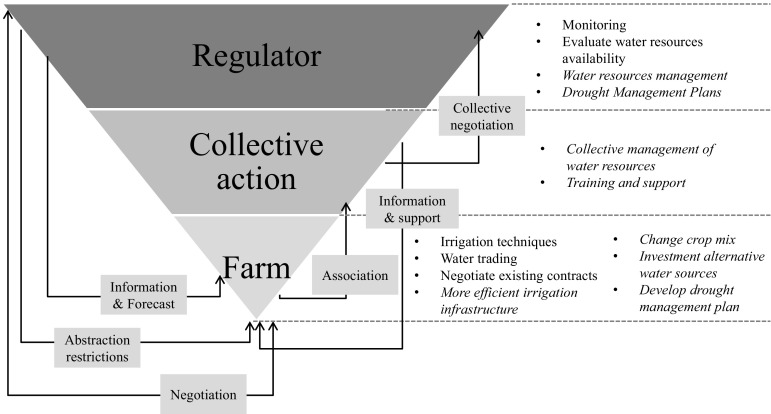
Main drought management actors and actions related to the agricultural sector at the different spatial scales (strategic planning activities shown in italics)

It is apparent, however, from the interview analyses that the decline in drought impacts is not just the result of investment in irrigation infrastructure, but of a range of actions at different scales. Figure 4 summarises the main actions and relationships amongst actors involved in agricultural drought management in Anglian region. Although based on our findings, the approach could equally apply to many other different contexts. For example, it highlights the importance of a vertically integrated drought management approach (Holman and Trawick 2011) for reducing the impacts on agriculture that considers not only on-farm responses (crucial for adapting to climate change and variability as stated by Reidsma et al. (2010)), but also how farmers work together to protect their interests and the pivotal role of the regulator to provide information and support. The key attributes of each level are outlined as follows:

- Farm: farmers are developing drought management plans so that high-value crops, drought-sensitive crops and forward contract commitments will be given priority for irrigation if there is insufficient water available. This is increasingly being combined with improved irrigation scheduling (Weatherhead and Rivas-Casado 2007) and water source diversification.
- Collective action at catchment scale: although water user associations or abstractor groups have existed for many decades in more arid countries, it is a relatively new phenomenon in the UK. Most of them were initiated in the 1990s after severe irrigation abstraction restrictions in the Anglian region (Leathes et al. 2008). They have facilitated dialogue between farmers and the EA and increased their power to better defend their water rights (Holman and Trawick 2011).
- Regulatory action at catchment to national scale: the water regulatory agency has significantly changed its relationship with the agricultural community in recent years. They are now considered to be much more proactive, providing better information and developing a partnership approach to drought management. This has been facilitated by local staff being given the flexibility, within the overall constraints of Drought Management Plans, to take both local catchment conditions and an understanding of agricultural needs and potential impacts into account when implementing drought management responses.

However, whilst there have been many positive developments in increasing drought resilience of irrigated agriculture, when we asked farmers about what areas of drought management should be improved, respondents identified a number of ongoing concerns:

1. As farmers played an important role in reducing the impacts of the 2010–2012 drought on aquatic ecosystems through voluntary restrictions, they would like to see a more collaborative approach to the management of catchment water resources, with stakeholders being partners in water resource and drought planning;
2. Seasonal forecasting of water availability needs to be improved to allow farmers to better plan for future weather- and water-related risks, as pointed out by many authors (Iglesias et al. 2003; Ramamasy and Baas 2007; Kgakatsi and de Rautenbach 2014);
3. There is a need for better reallocation of water resources within agriculture. Although a few respondents reported successful experiences trading water during recent drought periods, legislative barriers still make trading cumbersome and slow. Short-term exchanges are generally not feasible under current standard procedures due to the lack of transparency and the time required for approval (Cave 2009; Ofwat, Environment Agency 2008, 2009; Severn Trent Water 2011), so water trading is rare (Defra and Welsh Government 2014). Overcoming these limitations within ongoing water abstraction reform (Defra 2014) could substantially improve drought management in the region.
4. The burden of drought impacts needs to be borne more equally across all sectors (NFU 2014). The fact that agriculture in the UK only uses 1% of the total water abstraction and the increasing concern for food security weakens the argument for agriculture being the only sector subject to compulsory abstraction restrictions. However, environmental impacts of droughts and water scarcity cannot be ignored (especially under the Water Framework Directive requirements), so there is also a need for an improved evidence base of the impacts of abstraction on ecology and ecological resilience to drought to achieve a balance between environmental sustainability/aquatic ecological status (Acreman et al. 2008; Poff and Zimmerman 2010), food security and rural livelihoods.

Finally, whilst these insights relate to a particular region with a specific national legislative context, many of the issues identified are widely transferable to other regions internationally, not only humid or temperate areas but also more arid and semi-arid regions. As the drought management in England is currently undertaken at the regional level, a national study similar to the one presented here will allow for the comparison of drought impacts and adaptation options amongst different regions with very different agroclimatic and soil conditions, cropping patterns and agricultural businesses.

## Conclusions

Irrigators in eastern England have been affected by several drought episodes over the past 30 years experiencing, in some cases, mandatory abstraction bans. This research aimed to understand how drought management at farm to catchment scale has evolved over time in this region, and to identify improvements to decision-making for the future. Our analyses have shown how farmers have adapted their businesses, being more resilient to drought now than they were some decades ago, despite increasing water scarcity. This has arisen through investments in alternative water sources, improved farm drought planning, collective action and improved working relationships with the regulator during drought. In addition, the way the regulator manages drought has also improved, changing to a more proactive attitude, recognising the importance of irrigators being involved in drought management and providing better forecast information to guide farm-level decisions. The importance of this vertically integrated management approach to reducing drought impacts on agriculture is clear. The increased frequency of drought associated with climate change and increasing water scarcity will require further collaborative partnership-based approaches to water resource and drought management to share the impact burden more equitably between water users in the future.

## Electronic supplementary material

Below is the link to the electronic supplementary material.

Supplementary material 1 (DOCX 16 kb)

Supplementary material 2 (DOCX 28 kb)

Supplementary material 3 (DOCX 14 kb)

## Footnotes

1 Emergency variations of licences for spray irrigation purposes (Water Act 1991, S57, p 44): http://www.legislation.gov.uk/ukpga/1991/57/pdfs/ukpga_19910057_en.pdf.

2 CEH Drought portal: https://eip.ceh.ac.uk/droughts.

3 Headroom is defined here as the difference between the maximum volume that can be abstracted (as defined in the abstraction licence) and the volume that is actually abstracted, i.e. the proportion of the licence that has not been used in a given year.

4 A reform of the water abstraction licencing system is currently being designed in England and Wales (UK Water White Paper) (Defra 2011).
