# Supplementary material for: Developing drought resilience in irrigated agriculture in the face of increasing water scarcity
Source: Reg Environ Change. 2017 Feb 8;17(5):1527–40. doi: 10.1007/s10113-017-1116-6 (PMC6979716; doi:10.1007/s10113-017-1116-6)
Supplement: Supplementary file 1 — Supplementary material 1 (DOCX 16 kb) [file 10113_2017_1116_MOESM1_ESM.docx]

**Introduction**

1. **In the online survey you gave us some details about the crops that you grow, the size of your farm, etc., but could you tell us a little bit about your story in the farming business?**
   1. For how long have you been in this business?
   2. Always same crops? Changes from rainfed to irrigated agriculture?

*“The next questions are going to be related to past droughts in order to better understand how drought management can be improved to deal with future events…”*

**Memories from past droughts**

1. **You said that your business activity was affected by the ………. drought (the first drought he/she mentioned in the online survey). What else could you tell us about it?**
   1. Duration, timing, etc.
   2. Losses?
   3. Any management action?
2. **What memories would you like to share with us about other drought episodes that affected your business?**
   1. Which drought?
3. Duration, timing, etc.
4. Losses?
5. Any management action?
6. **I can see in the online survey that you suffered some abstraction restrictions during past droughts. We would like to know more about how Section 57 has affected your business?**
   1. How section 57 is applied? Triggers, amount of notice (is it transparent, fair, …?)
   2. Information for growers about restrictions
   3. Ability to negotiate with EA if restrictions/bans applied?
7. **Your production is mainly for ….. (processing/supermarkets/local farmers’ market/exports/other). How the impacts of a drought episode on crop production could have affected them and other actors in between?**
   1. Impacts on other agents of the supply chain
   2. Problems
   3. Ways of negotiating

*“Now, we are going to ask you a few questions related to drought management at the different levels, and ways of improvement…”*

**Drought management**

1. **You said in the online survey that your main strategies during a drought are…………Could you describe how is the decision process during/after a drought about which on-farm strategies to implement to cope with it?**
   1. How to choose among them?
   2. Triggers and timing for implementation?
2. **Would you consider that your attitude towards drought risk have changed after being affected by several drought episodes?**
   1. Any strategies at the beginning of the season (ex ante, prevention strategies), when you don’t know how the year is going to be (wet, dry)?
   2. Any long-term management actions taken? changes in crop mix (more resistant to drought), building reservoirs
3. **What would you do, in terms of drought management, if droughts (or abstraction restrictions) become more frequent and/or severe?**
   1. Changes in drought management strategies?
4. **How did governmental bodies and farmers associations support farmers in drought management and which aspects could be improved?**
   1. Environment Agency
   2. UKIA, NFU
   3. WAG
5. **How do you perceive drought management in the UK has evolved over time?**
   1. At the national level (related to agriculture)?
   2. At the regional/local level?
   3. At the farm level?
6. **In the online survey, you said that the water management aspects that should be changed to reduce the impacts of future droughts in the UK irrigated agriculture are: ……… Could you explain us why do you believe these are the most important issues to be improved and how can be achieved?**

**Final questions**

1. **In your opinion, what can be learned from previous drought episodes in the UK?**
   1. what things do you believe could have done better in relation to drought management in previous events?
2. **Would you like to add anything before concluding this interview?**
