# Supplementary material for: Developing drought resilience in irrigated agriculture in the face of increasing water scarcity
Source: Reg Environ Change. 2017 Feb 8;17(5):1527–40. doi: 10.1007/s10113-017-1116-6 (PMC6979716; doi:10.1007/s10113-017-1116-6)
Supplement: Supplementary file 2 — Supplementary material 2 (DOCX 28 kb) [file 10113_2017_1116_MOESM2_ESM.docx]

**Q1. Farm postcode:**

|  |
| --- |

**Q2. Farm size**

|  |  |
| --- | --- |
| Total farm size (ha) |  |
| Proportion that can be irrigated (in %) |  |
| Proportion that was irrigated in 2014 (in %) |  |

**Q3. Which crops do you grow and what are their average yields?**

*Please tick all that apply*

| Crops | Rainfed | Irrigated | Rainfed average yield (tonnes/ha) | Irrigated average yield (tonnes/ha) |
| --- | --- | --- | --- | --- |
| Maincrop potatoes |  |  |  |  |
| Early potatoes |  |  |  |  |
| Vegetables |  |  |  |  |
| Cereals |  |  |  |  |
| Sugar beet |  |  |  |  |
| Grass |  |  |  |  |
| Small fruit |  |  |  |  |
| Orchard fruit |  |  |  |  |

**Q4. From which water sources do you get the irrigation water from?**

*Please ensure the total =100*

|  | *%* |
| --- | --- |
| Surface Water (including ponds, lakes, gravel or clay workings, rivers, streams or other water course) |  |
| Ground Water (including wells, bore holes and springs rising on the holding) |  |
| Public mains water supply |  |
| Rainwater collected on site |  |
| Re-use of water from other purposes |  |
| Other (please specify): |  |
| ***TOTAL*** | ***100*** |

**Q5. What type of abstraction licence do you have for irrigating your crops?**

*Please tick all that apply*

- All year abstraction
- Winter-only abstraction
- Summer-only abstraction

**Q6. What irrigation method(s) do you use? Please, give the answer in % of irrigated area**

*Please ensure the total =100*

|  | *%* |
| --- | --- |
| Static or hand-moved sprinklers, spray lines |  |
| Hose reels with rain guns |  |
| Hose reels with booms |  |
| Centre pivots or linear moves |  |
| Trickle or drip |  |
| Other(s) (please specify): |  |
| ***TOTAL*** | ***100*** |

**Q7. How do you decide when and how much to irrigate?**

*Please ensure the total =100*

|  | *%* |
| --- | --- |
| Water balance calculation (by hand, computer) |  |
| In-field soil moisture measurement |  |
| Judgment (feeling the soil, crop inspection,...) |  |
| Other(s) (please specify): |  |
| ***TOTAL*** | ***100*** |

**Q8. What is the final destination for your products?**

*Please tick all that apply*

|  | Local farmers' market | Processing | Supermarket | Exports | Other |
| --- | --- | --- | --- | --- | --- |
| Maincrop potatoes |  |  |  |  |  |
| Early potatoes |  |  |  |  |  |
| Vegetables |  |  |  |  |  |
| Cereals |  |  |  |  |  |
| Sugar beet |  |  |  |  |  |
| Grass |  |  |  |  |  |
| Small fruit |  |  |  |  |  |
| Orchard fruit |  |  |  |  |  |

**Q9. Has your production (yield and/or quality) been affected by any drought episode over the last few decades?**

*Please tick the drought period(s) below that affected your business and estimate the impact level in each case*

|  | No impact | Low | Medium | High |
| --- | --- | --- | --- | --- |
| 1976 |  |  |  |  |
| 1988-1992 |  |  |  |  |
| 1995-1997 |  |  |  |  |
| 2003 |  |  |  |  |
| 2004-2006 |  |  |  |  |
| 2010-2012 |  |  |  |  |
| Other event (please specify the year(s)): |  |  |  |  |

**Q10. For the most recent drought episode in which your business was affected, what was the typical yield reduction compared with average yield for your most important crops?**

*Please provide an estimate in % or tonnes per ha, whichever is easiest*

| Crop type | Yield reduction (%) | Yield reduction (tonnes/ha) |
| --- | --- | --- |
|  |  |  |
|  |  |  |
|  |  |  |

**Q11. Similarly, for the most recent drought episode, what were the typical impacts on farm-gate prices?**

|  | No effect | Slight increase in price (less than 5%) | Moderate increase in price (5 to 10%) | High increase in price (more than 10%) | Slight decrease (less than 5%) | Decrease of more than 5% |
| --- | --- | --- | --- | --- | --- | --- |
| Maincrop potatoes |  |  |  |  |  |  |
| Early potatoes |  |  |  |  |  |  |
| Vegetables |  |  |  |  |  |  |
| Cereals |  |  |  |  |  |  |
| Sugar beet |  |  |  |  |  |  |
| Grass |  |  |  |  |  |  |
| Small fruit |  |  |  |  |  |  |
| Orchard fruit |  |  |  |  |  |  |

**Q12. During/after a drought, did you experience any contractual/supply problems?**

- No problem experienced
- Yes, I achieved a lower price for my product
- Yes, I did not achieve the agreed production as stipulated in the contract
- Yes, because of quality issues
- Yes, other reason(s) (please specify): ………………………………………………………

**Q13. Have you experienced any abstraction restrictions during recent droughts?**

|  | Yes, voluntary restrictions | Yes, mandatory restrictions | Yes, mandatory bans | No restriction |
| --- | --- | --- | --- | --- |
| 1976 |  |  |  |  |
| 1988-1992 |  |  |  |  |
| 1995-1997 |  |  |  |  |
| 2003 |  |  |  |  |
| 2004-2006 |  |  |  |  |
| 2010-2012 |  |  |  |  |
| Other event (please specify the year(s)): |  |  |  |  |

**Q14. What source(s) of information did you use during the most recent drought to raise your awareness?**

- TV/Radio
- Newspaper
- Met Office website
- Environment Agency website
- Grower levy board (AHDB)
- Producer organizations (PO)
- Other source(s) (please specify): …………………………………………………………..

**Q15. What strategies do you implement when a drought has been declared and abstraction restrictions are likely?**

*Please tick any of the actions that are relevant to you in the left-hand column.*

*In the right-hand column, please tick two actions that you consider to be of highest importance.*

|  |  | Top 2 |
| --- | --- | --- |
| Abstract to maximum to get soil water contents up |  |  |
| Irrigated a reduced area to their full irrigation schedule |  |  |
| Irrigate the full area to a reduced irrigation schedule |  |  |
| Irrigate at night |  |  |
| Renegotiate existing supply contracts |  |  |
| Develop a drought management plan |  |  |
| Evaluate water resource position |  |  |
| Personally negotiate with EA |  |  |
| Work with local Water Abstractor Group to negotiate with EA |  |  |
| Seek informal water trades |  |  |
| Other(s) (please specify): |  |  |

**Q16. After the last drought episode, did your business make any changes in farm management to cope with future drought risks?**

- No
- Yes, investment in alternative water sources to reduce risk (e.g. reservoir)
- Yes, changes in the crop mix or varieties to make them more resistant to droughts
- Yes, development of a business drought plan
- Yes, other change(s) (please specify):…………………………………………………………

**Q17. In your opinion, what water management aspects could be changed to reduce the impacts of future droughts on the UK irrigated agricultural sector?**

- Removal of Section 57 restrictions
- Implement a national crop insurance system
- Encourage water trading within the agricultural sector
- Improve information and forecasting of drought impacts for farming community
- Give farming sector a more central role in catchment water management
- Other(s) (please specify): …………………………………………………………………..

**Q18. On a scale of 0 to 10, how do you rate drought risk to your farm business? 0 is ’not important at all’, and 10 is ‘very important’.**

| - 0 | - 3 | - 6 | - 9 |
| --- | --- | --- | --- |
| - 1 | - 4 | - 7 | - 10 |
| - 2 | - 5 | - 8 |  |

**Q19. Do you think droughts and water scarcity are likely to become more frequent in the future in the UK?**

- No
- Not sure
- Very unlikely
- Likely
- Highly likely

**Q20. Most growers are willing to invest in drought risk management strategies, but which options are more relevant to your business?**

- No, not willing to invest in drought management strategies
- Yes, modernization of irrigation equipment is a priority
- Yes, an on-farm reservoir provides the greatest security
- Yes, changing soil and crop management practices (tied-ridging, mulching, better soil management, switching to more drought tolerant varieties)
- Yes, other(s) (please specify): ………………………………………………………………..

**If you have any other specific comments relating to drought impacts on your farm or policy actions needed then please provide further information below**

|  |
| --- |

**Please provide your contact details if you wish to receive a summary of the results or are willing to further contribute to these projects.**

*This data will be used only for these purposes. Your answers will be still anonymous*

- I would like to receive a summary of the results
- I am willing to further contribute to these projects (You may be contacted in order to know more about your experience dealing with droughts).

| First name |  |
| --- | --- |
| Last name |  |
| Email address |  |
| Mobile number |  |
