# Supplementary material for: Developing drought resilience in irrigated agriculture in the face of increasing water scarcity
Source: Reg Environ Change. 2017 Feb 8;17(5):1527–40. doi: 10.1007/s10113-017-1116-6 (PMC6979716; doi:10.1007/s10113-017-1116-6)
Supplement: Supplementary file 3 — Supplementary material 3 (DOCX 14 kb) [file 10113_2017_1116_MOESM3_ESM.docx]

**Thematic coding - Final template**

1. DROUGHT
   1. Definition and risk perception
   2. Past event
      1. 1976
      2. 1988-1992
      3. 1995-1997
      4. 2003
      5. 2004-2006
      6. 2010-2012
   3. Future risk
2. DROUGHT MANAGEMENT AND RESPONSE
   1. Environment Agency
      1. Support and information
      2. Section 57
      3. Environmental protection
   2. On-farm
      1. Crop prioritization
      2. Change crop mix
      3. Reservoirs
      4. Water trading
      5. Based on contract commitments
      6. Develop a drought management plan
      7. Personally negotiate with the EA
      8. WAG
      9. Evaluate water resource position
      10. Abstract to a maximum to get soil water content up
      11. Irrigate reduced area to the full schedule
      12. Irrigate full area to a reduced schedule
      13. Irrigate at night
      14. Others
   3. Food supply chain
   4. Areas for improvement
